# Low skull bone density is associated with poor motor prognosis in women with Parkinson’s disease

**DOI:** 10.3389/fnagi.2022.1053786

**Published:** 2022-11-15

**Authors:** Seong Ho Jeong, Namki Hong, Hye Sun Lee, Sookyeong Han, Young-gun Lee, Yoonju Lee, Yumie Rhee, Young H. Sohn, Phil Hyu Lee

**Affiliations:** ^1^Department of Neurology, Yonsei University College of Medicine, Seoul, South Korea; ^2^Department of Neurology, Sanggye Paik Hospital, Inje University College of Medicine, Seoul, South Korea; ^3^Department of Internal Medicine, Severance Hospital, Endocrine Research Institute, Yonsei University College of Medicine, Seoul, South Korea; ^4^Biostatistics Collaboration Unit, Yonsei University College of Medicine, Seoul, South Korea; ^5^Endocrine Research Institute, Severance Hospital, Seoul, South Korea; ^6^Department of Health Promotion, Severance Health Check-Up, Severance Hospital, Yonsei University Health System, Seoul, South Korea; ^7^Severance Biomedical Science Institute, Yonsei University College of Medicine, Seoul, South Korea

**Keywords:** Parkinson’s disease, osteopenia/osteoporosis, dopamine transporter (DAT) imaging, prognosis, skull bone density

## Abstract

Parkinson’s disease (PD) and osteoporosis are degenerative diseases that have shared pathomechanisms. To investigate the associations of skull bone density with nigrostriatal dopaminergic degeneration and longitudinal motor prognosis in female patients with PD. We analyzed the data of 260 drug-naïve female PD patients aged ≥50 years old who were followed-up for ≥3 years after their first visit to the clinic with baseline dopamine transporter (DAT) imaging. We measured skull bone density as a surrogate marker for systemic bone loss by calculating the Hounsfield unit (HU) in computed tomography scans. A Cox proportional hazard model was built to compare the rates of levodopa-induced dyskinesia (LID) or wearing-off according to skull HU. Longitudinal changes in levodopa-equivalent dose (LED) during a 3-year follow-up were assessed using a linear mixed model. A lower skull HU was associated with lower baseline DAT availability in striatal subregions; however, this relationship was not significant after adjusting for age, disease duration, body mass index, and white matter hyperintensities. After adjusting for confounding factors, a lower skull HU was significantly associated with an increased risk of LID development (hazard ratio = 1.660 per 1 standard deviation decrease, *p* = 0.007) and wearing-off (hazard ratio = 1.613, *p* = 0.016) in younger (<67 years) but not in older patients. Furthermore, a lower skull HU was associated with a steeper increase in LED during follow-up in younger patients only (β = –21.99, *p* < 0.001). This study suggests that baseline skull bone density would be closely linked to motor prognosis in drug naïve women with PD.

## Introduction

Parkinson’s disease (PD) is the second most common neurodegenerative disorder, characterized by the progressive loss of dopaminergic neurons in the substantia nigra of the midbrain (Braak et al., 2003). Aging is the most important risk factor for PD due to its close relation to PD pathogenesis and its contribution to disease progression (Collier et al., 2017; Lee et al., 2017).

Osteoporosis is a common systemic skeletal disease characterized by decreased bone mass and strength, leaving one susceptible to fragility fracture as a result of the aging process (Hernlund et al., 2013). One important feature of osteoporosis is the contribution of sex as a risk factor, in which the prevalence of osteoporosis is four times greater in women than in men (Alswat, 2017). Ample evidence has demonstrated an association between osteoporosis and PD. Osteoporosis is prevalent in PD patients, and accordingly, PD patients have a higher risk of osteoporosis and osteoporotic fractures (Torsney et al., 2014). A recent study has demonstrated that osteoporosis is a risk factor for the development of PD among women (Feng et al., 2021). This association may be ascribed to overlapping pathomechanisms, such as autophagy dysfunction, mitochondrial dysfunction and inflammation (Wang et al., 2016; Wang et al., 2020). Another common feature underpinning these two conditions is the effect of sex hormones. Specifically, menopause has been reported risk factor of both osteoporosis and PD (Ragonese et al., 2006; Ji and Yu, 2015), and postmenopausal estrogen use prevents the development of both diseases (Syed et al., 2008; Lee et al., 2019).

Until now, the effects of bone mass on nigrostriatal dopaminergic degeneration and longitudinal motor prognosis in PD patients have not yet been investigated. Conveniently, Hounsfield unit (HU) values of the skull bone can be obtained from computed tomography (CT) scans performed concurrently with dopamine transporter (DAT) imaging. Moreover, decreased skull bone HU has been proposed as a surrogate marker of systemic bone loss, which can be utilized as a tool for opportunistic osteoporosis screening (Na et al., 2018). In this study we hypothesized that low skull bone HU can predict poor long-term motor prognosis in female drug naïve PD patients. We performed an analysis of striatal DAT availability and clinical parameters of longitudinal disease progression, including longitudinal increases in dopaminergic medication doses and motor complication rates in female PD patients, all according to the severity of skull bone density loss.

## Materials and methods

### Participants

We retrospectively reviewed 660 *de novo* PD patients who were drug-naïve and visited the Movement Disorders outpatient clinic at the Severance Hospital from April 2009 to September 2015. We used the following inclusion criteria: (1) female sex, (2) age at diagnosis ≥50 years, and (3) follow-up duration ≥3 years. The cut-off value of age at diagnosis in this study was based on the median age of menopause in Korean women (Park et al., 2018), and PD was diagnosed according to the clinical diagnostic criteria of the United Kingdom Parkinson’s disease Society Brain Bank. Additionally, *N*-(3-[^18^F]fluoropropyl)-2β-carbon ethoxy-3β-(4-iodophenyl) nortropane positron emission tomography (^18^F-FP-CIT PET) scans revealed decreased DAT availability in the posterior putamen of all patients. Parkinsonian motor symptoms were assessed at initial visit under dopaminergic drug-naïve condition using the Unified Parkinson’s Disease Rating Scale Part III (UPDRS-III) (Park et al., 2020), and olfactory function was measured using the cross-cultural smell identification test (CCSIT). Depression was evaluated using the Beck Depression Inventory (BDI), and the Korean version of the Mini-Mental State Examination (MMSE) was used to assess general cognition among patients (Han et al., 2008). In the final cohort, we included 260 female *de novo* PD patients, excluding (1) 316 male patients, (2) 66 patients with follow-up duration <3 years, (3) 13 patients with age at diagnosis <50 years, and (4) 5 patients with imaging process error. All enrolled patients were followed-up for a mean period of 6.22 ± 1.99 years and did not present with additional atypical features, including poor response to dopaminergic medications, ataxia, prominent autonomic dysfunction, vertical gaze limitation, repeated unprovoked falls within 3 years of parkinsonian symptom onset, and cortical sensory loss during the follow-up period. All patients were also investigated for medical history and vascular risk factors, including hypertension, diabetes mellitus, dyslipidemia, cardiac disease, and ischemic stroke.

### Acquisition and quantitative analyses of ^18^F-FP-CIT PET scans

^18^F-FP-CIT PET scans were acquired using a GE PET-CT DSTe scanner (GE Discovery STE; GE Healthcare; Milwaukee, WI, USA), which obtains images with a three-dimensional resolution of 2.3-mm full width at half maximum. After fasting for ≥6 h, the patients were intravenously injected with 5 mCi (185 MBq) of ^18^F-FP-CIT. 90 min after the injection, PET images were acquired for 20 min in the three-dimensional mode at 120 kVp and 380 mA, and image processing was performed using the SPM8 software (Wellcome Department of Imaging Neuroscience, Institute of Neurology, UCL, London, UK) with Matlab 2013a for Windows (Math Works, Natick, MA, USA). Quantitative analyses in this procedure were based on volumes of interests (VOIs), which were defined based on a template in standard space. After imaging processing, all reconstructed PET images were spatially normalized into the Montreal Neurology Institute template space using a standard ^18^F-FP-CIT PET template which was generated from ^18^F-FP-CIT PET and T1-weighted magnetic resonance imaging (MRI) scans of 13 normal controls. Twelve VOIs of bilateral striatal subregions and one occipital VOI were drawn on a co-registered, spatially normalized, single T1-weighted MR and ^18^F-FP-CIT PET template image on the MRIcro version 1.37 software (Chris Rorden, Columbia, SC, USA) (Oh et al., 2012). The striatum was divided along the anterior-posterior commissure line on the transverse plane into dorsal and ventral portions. The ventral portion comprised two subregions: the ventral putamen and ventral striatum. Meanwhile, the dorsal portion was divided along the coronal anterior commissure plane into the following anterior and posterior subregions: the anterior caudate, posterior caudate, anterior putamen, and posterior putamen. These VOIs were adjusted using a minor translation in our in-house editing software, ANIQUE (Oh et al., 2014). DAT availability was calculated by the non-displaceable binding potential, which was defined as follows: (mean standardized uptake value of the striatal subregions VOI–mean standardized uptake value of the occipital VOI)/(mean standardized uptake of the occipital VOI) (Innis et al., 2007).

### Skull bone Hounsfield unit measurement

**FIGURE 1:**
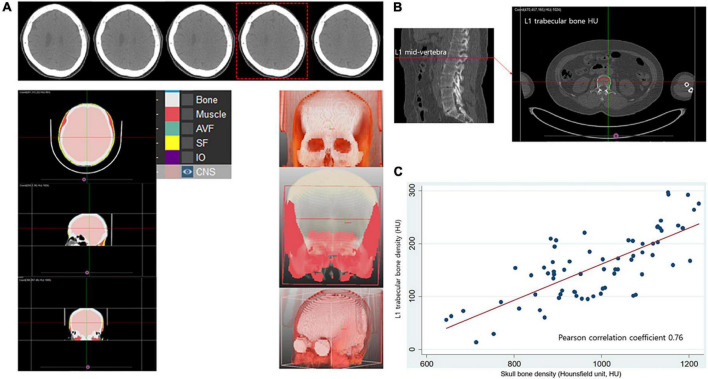
Measurement of Hounsfield unit in skull and lumbar bone and their correlation in healthy control. **(A)** Segmentation of skull bone and calculation of radiation attenuation value of skull bone area using automated deep learning software (DeepCatch, Medical IP, Korea). Skull bone HU values were measured using single slice CT image at the predefined level where the lateral ventricles immediately disappear. **(B)** Measurement of lumbar spine trabecular bone radiation attenuation. **(C)** Significant positive correlation (*r* = 0.76, *p* < 0.001) between skull bone density and lumbar spine trabecular bone density measured from computed tomography scans. HU, Hounsfield unit.

Skull bone HU was measured using a single slice CT image at the predefined level where the lateral ventricles immediately disappear based on prior studies (Figure 1A; Na et al., 2018). To calculate skull bone HU from the entire skull area, a commercially available deep learning software, which can segment bone and soft tissues based on the 3D U-Net model was used (DeepCatch ver. 1.0.0.0, MEDICALIP Co. Ltd., Seoul, Korea) (Kim et al., 2021; Lee et al., 2021). To train the model for bone segmentation deployed in DeepCatch software, threshold of 450 HU, which was known to minimized differences of cortical bone estimate between higher resolution CT and histological methods in prior reports, (Poole et al., 2010; Treece et al., 2010) was applied to crudely segment outline of bone mask in each CT slice, followed by manual correction by expert radiologist with more than 10 years of experience. After applying DeepCatch 3D U-Net model to segment the skull bone mask, human inspection was performed with manual correction for the masks if required. Semi-automatic segmentation performance for the bone mask measured using the Dice similarity coefficient was calculated to be 98.6 ± 1.1% (a degree of overlap between U-Net-driven masks and reference masks, *p* < 0.001), and inter-rater reliability of skull bone HU measurement using the 3D U-Net model was noted to be good (intraclass correlation 0.99, *p* < 0.001) when assessed in 30 randomly sampled skull CT images. To determine whether skull bone density can reflect systemic bone loss in women, we evaluated the correlation between skull bone and lumbar spine HU in a separate health examination cohort, comprising community-dwelling individuals (Severance Health Check-Up cohort), which was approved by the Institutional Review Board of Severance Hospital (IRB No. 4-2021-0524). In that study, 77 women underwent an individual health examination package, including a ^18^F-fluorodeoxyglucose PET-CT scan of the whole body and brain performed at the same day in a commercial examination center (Severance Health Check-Up Center, Seoul, South Korea) between 2010 and 2020. After excluding those with ethnicities other than Korean and those <40 years of age, CT scans of 70 individuals were retrospectively analyzed (Siemens Biograph 40 TruePoint PET-CT, 120 kVp). Lumbar spine HU was then measured at the manually positioned ellipsoid region of interest in the mid-vertebral level of first lumbar spine (L1) using the MEDIP PRO software (Medical IP, Korea), a well validated method for CT-based opportunistic osteoporosis screening (Figure 1B; Jang et al., 2019). The skull bone and L1 HU showed a good positive correlation (Pearson correlation coefficient = 0.76, *p* < 0.001; Figure 1C).

### Acquisition of fluid attenuated inversion recovery sequence images and grading of white matter hyperintensities

Of the 260 enrolled patients, 201 (77.3%) underwent brain MRI scans at the Severance Hospital using a 3.0 T scanner (Achieva; Philips Medical System, Best, Netherlands) at initial assessment, which included fluid attenuated inversion recovery (FLAIR) sequence images. The FLAIR sequence images were acquired with the following parameters: matrix, 512 × 512; slice number, 22; pixel spacing, 0.449 mm × 0.449 mm; slice thickness, 5 mm; gap, 2 mm; field of view, 230 mm; repetition time, 11,000 ms; echo time, 125 ms; inversion time, 2,800 ms; and flip angle, 90°. The remaining 59 (22.7%) patients underwent brain MRI scans with FLAIR sequence images at other hospitals before being referred to Severance Hospital. The visual rating scale of the white matter hyperintensities (WMHs) was assessed by two neurologists (SHJ and Y-gL) using the Scheltens scale, wherein the periventricular and lobar (frontal, parietal, temporal, and occipital) white matter hyperintensities, and the basal ganglia and infratentorial signal hyperintensities were rated separately in a semi-quantitative manner (Scheltens et al., 1993). The intra- and inter-rater reliability of total WMHs were high (intra class correlation coefficients = 0.984 and 0.966, respectively). A final consensus rating between the two raters was used for subsequent analyses.

### Assessment of the development of levodopa-induced dyskinesia and wearing-off

Participants visited our outpatient clinic every 3–6 months, and two movement disorder experts (YHS and PHL) carefully assessed the presence of LID and wearing-off through a history obtained from the patients and caregivers or direct neurological examination at every visit (Jeong et al., 2021a). For consistency, the date on which the female PD patients or their caregivers reported LID or wearing-off occurrence or the date on which LID or wearing-off was first observed in the clinic was regarded as the date of LID or wearing-off occurrence in the medical records. To assess the effects of skull HU on the development of LID and wearing-off, the Cox regression model was used to estimate the hazard ratio (HR) and 95% confidence intervals (CIs), while adjusting for age at diagnosis, disease duration, baseline DAT availability in the posterior putamen, total WMHs, BMI, and levodopa equivalent dose (LED) at LID or wearing-off onset in patients with LID or wearing-off or LED at the last visit to the outpatient clinic in those without either.

### Longitudinal assessment of the changes in levodopa-equivalent daily doses over time

The patients visited the outpatient clinic every 3–6 months and their dopaminergic medications were adjusted for effective symptom control by PHL and YHS according to their individual responses. At each visit, dopaminergic medication doses were assessed, and the LED was calculated using the following formula: levodopa × 1 + controlled-release levodopa × 0.75 + ropinirole × 20 + pramipexole × 100 + levodopa × 0.33 if they were on entacapone + selegiline × 10 + rasagiline × 100 (Tomlinson et al., 2010). Thereafter, a linear mixed model was used to compare the rate of longitudinal changes in LED for 3 years, and participants were added as random effects, whereas age at diagnosis, disease duration, and DAT availability in the posterior putamen, BMI, and total WMHs were added as fixed effect terms. The effect of the skull HU on longitudinal changes in LED was tested using a skull HU × time interaction term.

### Statistical analyses

To investigate the effect of skull HU per a standard deviation (SD), we used individual z-transformed skull HU values in all analyses. A multivariate linear regression analysis was used to determine the independent effects of skull HU after adjusting for age at diagnosis, disease duration, BMI, and total WMHs, a linear mixed model was used to compare the rates of the longitudinal LED changes according to skull HU, and a log-rank test and Cox regression model were used to assess the effects of skull HU on the development of LID and wearing-off. Since age is a crucial factor for osteoporosis, we further investigated whether an age × skull HU interaction term was significant in multivariate regression, Cox regression, and linear mixed model analyses, wherein an interaction term with *p* < 0.2 was considered an interaction effect (Jeong et al., 2021b). If an interaction term was significant, we further performed subgroup analyses using the median split method to dichotomize PD patients into the young and old age groups. Statistical analyses were performed using the STATA 16.1 (StataCorp LLC, College Station, TX, USA) and R (v4.0)^1^ software, and results with a two-tailed *p* < 0.05 were considered statistically significant.

## Results

### Demographic and clinical characteristics

Demographic and clinical characteristics of the included patients are summarized in Table 1. Age at symptom onset and diagnosis was 64.58 ± 8.58 and 66.11 ± 8.49, respectively. The average disease duration was 17.14 ± 16.98 months, UPDRS-III score at the time of PD diagnosis was 22.72 ± 10.22, average years of education was 7.92 ± 4.16 years, and MMSE score at baseline was 26.34 ± 2.95. Additionally, the average CCSIT and BDI scores were 7.09 ± 2.22 and 13.66 ± 8.75, respectively, and the average skull bone HU was 709.88 ± 123.96.

**TABLE 1:** Demographic characteristics.

Female patients with PD (*n* = 260)	
Age at PD onset (years), mean ± SD	64.58 ± 8.58
Age at diagnosis (years), mean ± SD	66.11 ± 8.49
Disease duration (months), mean ± SD	17.14 ± 16.98
UPDRS-III, mean ± SD	22.72 ± 10.22
Years of education (years), mean ± SD	7.92 ± 4.16
K-MMSE, mean ± SD	26.34 ± 2.95
CCSIT, mean ± SD	7.09 ± 2.22
BDI, mean ± SD	13.66 ± 8.75
**Vascular risk factors**	
Hypertension, n (%)	102 (39.2%)
Diabetes mellitus, n (%)	41 (15.8%)
Dyslipidemia, n (%)	50 (19.2%)
Cardiac disease, n (%)	23 (8.9%)
Ischemic stroke, n (%)	7 (2.7%)
BMI, mean ± SD	23.19 ± 3.12
Total WMHs, mean ± SD	10.58 ± 7.97
**DAT availability**	
Anterior caudate, mean ± SD	2.26 ± 0.68
Posterior caudate, mean ± SD	1.52 ± 0.56
Anterior putamen, mean ± SD	2.36 ± 0.64
Posterior putamen, mean ± SD	1.46 ± 0.48
Ventral putamen, mean ± SD	1.55 ± 0.43
Ventral striatum, mean ± SD	2.19 ± 0.57
Skull HU, mean ± SD	709.88 ± 123.96

BDI, Beck Depression Inventory; BMI, body mass index; CCSIT, Cross-Cultural Smell Identification Test; HU, Hounsfield units; MMSE, mini-mental status examination; UPDRS III, Unified Parkinson’s Disease Rating Scale Part III; WMHs, white matter hyperintensities.

### Association between skull Hounsfield unit and striatal dopamine transporter availability at baseline

Dopamine transporter availability for each striatal sub-region is shown in Table 1. The lower skull HU was associated with lower baseline DAT availability in the anterior caudate (β = −0.176, SE = 0.041, *p* < 0.001), posterior caudate (β = −0.137, SE = 0.034, *p* < 0.001), anterior putamen (β = −0.114, SE = 0.039, *p* = 0.004), and ventral striatum (β = −0.111, SE = 0.035, *p* = 0.001). However, these associations were not statistically significant after adjustment for age at diagnosis, disease duration, BMI, and total WMHs. Furthermore, the age × skull HU interaction term in each striatal sub-region was not significant (Table 2).

**TABLE 2:** Multiple linear regression analysis of dopamine transporter availability in each striatal subgroup.

Striatal subgroup	Anterior caudate		Posterior caudate		Anterior putamen		Posterior putamen		Ventral putamen		Ventral striatum	
	β (SE)	*p*	β (SE)	*p*	β (SE)	*p*	β (SE)	*p*	β (SE)	*p*	β (SE)	*p*
Univariate model												
Skull HU per 1 SD decrease	−0.176 (0.041)	<0.001	−0.137 (0.034)	<0.001	−0.114 (0.039)	0.004	−0.020 (0.030)	0.508	−0.034 (0.027)	0.201	−0.111 (0.035)	0.001
Multivariable model*												
Skull HU per 1 SD decrease	−0.027 (0.042)	0.517	−0.036 (0.037)	0.327	−0.037 (0.044)	0.399	0.005 (0.035)	0.883	0.017 (0.030)	0.579	−0.011 (0.037)	0.768
Interaction model*												
Skull HU per 1 SD decrease	−0.221 (0.280)	0.431	−0.275 (0.244)	0.261	0.270 (0.295)	0.361	0.242 (0.501)	0.293	0.117 (0.199)	0.557	0.086 (0.152)	0.727
Age × Skull HU	0.003 (0.004)	0.373	0.005 (0.004)	0.199	−0.004 (0.004)	0.427	−0.004 (0.003)	0.279	−0.002 (0.003)	0.499	−0.001 (0.004)	0.756

*Results of multivariate linear regression analysis for the striatal subgroup after controlling for age at diagnosis, disease duration, BMI, and total WMHs.

β, regression coefficient; BMI, body-mass index; HU, Hounsfield units; WMHs, white matter hyperintensities.

### Effect of skull Hounsfield unit on the development of levodopa-induced dyskinesia and wearing-off

During the follow-up period, LID and wearing-off developed in 83 (31.9%) and 75 (28.8%) of the 260 female PD patients, respectively. After adjusting for age at diagnosis, disease duration, BMI, total WMHs, and LED, lower skull HU tended to be associated with greater risks of developing LID (HR = 1.251 per 1 SD decrease of skull HU, 95% CI = 0.978–1.599, *p* = 0.074), whereas no significant association between baseline skull HU and the development of wearing-off was noted (HR = 1.142, 95% CI = 0.878–1.484, *p* = 0.321, Table 3). Since age × skull HU interaction terms were significant in the LID (*p* = 0.138) and wearing-off (*p* = 0.134) models, we performed subgroup analyses using the median split of age (median age = 67). Additionally, after adjusting for covariates, the age group × skull HU interaction terms were also found to be significant (LID, *p* = 0.164; wearing-off, *p* = 0.066). In the subgroup analyses, skull HU was a significant predictor for the development of LID (HR = 1.660, 95% CI = 1.150–2.398, *p* = 0.007) and wearing-off (HR = 1.613, 95% CI = 1.094–2.380, *p* = 0.016) in the young age group. However, there was no significant association between skull HU and the development of LID (HR = 1.019, 95% CI = 0.705–1.473, *p* = 0.919) or wearing-off (HR = 0.845, 95% CI = 0.565–1.268, *p* = 0.418) in the old age group (Table 4).

**TABLE 3:** Multivariate Cox hazard models for prediction of LID and wearing-off.

Variables	Development of LID				Development of wearing-off			
	C-index = 0.664		C-index = 0.666		C-index = 0.695		C-index = 0.705	
	Hazard ratio (95% CI)	*p*	Hazard ratio (95% CI)	*p*	Hazard ratio (95% CI)	*p*	Hazard ratio (95% CI)	*p*
Age at diagnosis	0.966 (0.936–0.996)	0.029	0.966 (0.937–0.996)	0.025	0.968 (0.937–1.001)	0.057	0.966 (0.935–0.997)	0.033
Disease duration	1.004 (0.991–1.018)	0.520	1.005 (0.992–1.019)	0.448	1.002 (0.987–1.018)	0.784	1.003 (0.988–1.019)	0.713
DAT availability, PP	0.744 (0.452–1.224)	0.244	0.776 (0.473–1.273)	0.315	1.106 (0.678–1.805)	0.686	1.137 (0.700–1.846)	0.604
WMHs	1.039 (1.010–1.069)	0.007	1.041 (1.013–1.071)	0.005	1.020 (0.988–1.054)	0.230	1.022 (0.990–1.056)	0.179
BMI	0.969 (0.903–1.040)	0.380	0.959 (0.893–1.031)	0.257	0.934 (0.865–1.008)	0.080	0.925 (0.856–1.000)	0.050
LED	1.001 (1.000–1.001)	0.083	1.001 (1.000–1.001)	0.125	1.002 (1.001–1.002)	<0.001	1.002 (1.001–1.002)	<0.001
Skull HU per 1 SD decrease	1.251 (0.978–1.599)	0.074	4.786 (0.800–28.642)	0.086	1.142 (0.878–1.484)	0.321	5.003 (0.713–35.127)	0.105
Age × Skull HU	–	–	0.979 (0.953–1.007)	**0.138**			**0.977 (0.948–1.007)**	**0.134**

Results of Cox regression analysis for the development of LID after controlling for age at diagnosis, disease duration, DAT availability in the posterior putamen, WMHs, BMI, and LED. BMI, body-mass index; DAT, dopamine transporter; HU, Hounsfield units; PP, posterior putamen; LED, levodopa-equivalent doses; LID, levodopa-induced dyskinesia; WMHs, white matter hyperintensities. Bold indicates *p*-value for interaction term < 0.2.

**TABLE 4:** Multivariate Cox hazard models for prediction of LID and wearing-off according to age subgroup.

Variables	Young age (<67 years, *n* = 123)				Old age (≥67 years, *n* = 137)			
	
	Development of LID		Development of wearing-off		Development of LID		Development of wearing-off	
	C-index = 0.721		C-index = 0.775		C-index = 0.669		C-index = 0.663	
	Hazard ratio (95% CI)	*p*	Hazard ratio (95% CI)	*P*	Hazard ratio (95% CI)	*p*	Hazard ratio (95% CI)	*p*
Age at diagnosis	0.941 (0.876–1.012)	0.102	0.944 (0.877–1.015)	0.120	0.988 (0.915–1.067)	0.764	0.943 (0.861–1.034)	0.214
Disease duration	1.005 (0.984–1.027)	0.642	1.005 (0.982–1.029)	0.658	1.007 (0.989–1.026)	0.431	1.001 (0.980–1.023)	0.935
DAT availability, PP	0.975 (0.490–1.942)	0.943	1.261 (0.641–2.480)	0.502	0.514 (0.238–1.111)	0.091	0.791 (0.364–1.718)	0.554
WMHs	1.037 (0.988–1.082)	0.147	1.006 (0.953–1.061)	0.836	1.053 (1.011–1.096)	0.013	1.031 (0.987–1.078)	0.170
BMI	0.896 (0.802–1.000)	0.050	0.855 (0.754–0.970)	0.015	1.019 (0.921–1.127)	0.720	0.980 (0.879–1.092)	0.716
LED	1.000 (0.999–1.001)	0.557	1.001 (1.001–1.002)	<0.001	1.001 (1.000–1.002)	0.155	1.002 (1.000–1.003)	0.022
Skull HU per 1 SD decrease	**1.660 (1.150–2.398)**	**0.007**	**1.613 (1.094–2.380)**	**0.016**	1.019 (0.705–1.473)	0.919	0.845 (0.565–1.268)	0.418

Results of Cox regression analysis for the development of LID after controlling for age at diagnosis, disease duration, DAT availability in the posterior putamen, WMHs, BMI, and LED. BMI, body-mass index; DAT, dopamine transporter; HU, Hounsfield units; PP, posterior putamen; LED, levodopa-equivalent doses; LID, levodopa-induced dyskinesia; WMHs, white matter hyperintensities. Bold indicates *p* < 0.05.

### Longitudinal assessment of the changes in levodopa-equivalent doses among the groups

**FIGURE 2:**
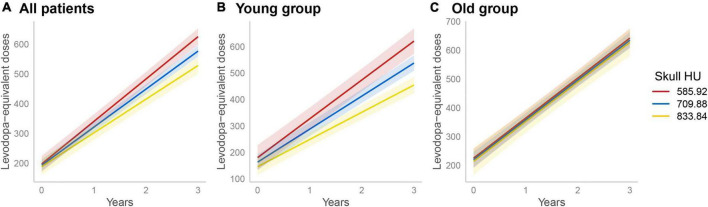
Longitudinal increases in levodopa-equivalent doses. A linear mixed model analysis showed significant difference in longitudinal LED increase according to skull HU values (skull HU per 1 SD decrease × Time, *p* < 0.001) **(A)**. In the subgroup analysis, longitudinal LED increase significantly differed according to skull HU in the young age group **(B)**, but not in the old age group **(C)**. The light red, blue, or yellow band represents the 95% confidence interval in each point of skull HU. LED, levodopa-equivalent doses; HU, Hounsfield unit; SD, standard deviation.

All enrolled PD patients were treated with dopaminergic medications for at least 3 years. In the linear mixed model, the skull HU × time interaction term was statistically significant (β = 14.66, SE = 3.42, *p* < 0.001) after adjusting for age at diagnosis, disease duration, DAT availability in the posterior putamen, BMI, total WMHs, skull HU, time, and skull HU × time, indicating that patients with low skull HU value had a rapidly increased LED (Figure 2A). Moreover, since age × skull HU × time interaction terms were significant in this model (*p* = 0.008, Table 5), we performed subgroup analyses using the median split of age. In the subgroup analyses, skull HU was a significant predictor for rapid LED increase over time in the younger subgroup (β = 21.99, SE = 4.41, *p* < 0.001, Figure 2B), whereas no significant association between skull HU and longitudinal LED increase was observed in the old age group (β = 0.70, SE = 5.96, *p* = 0.907, Figure 2C).

**TABLE 5:** Longitudinal changes of levodopa-equivalent doses.

Variables	Model 1		Model 2		Young age (<67 years)		Old age (≥67 years)	
	β (SE)	*p*	β (SE)	*p*	β (SE)	*p*	β (SE)	*p*
Age at diagnosis	2.53 (1.07)	0.018	2.40 (1.22)	0.049	−0.23 (2.37)	0.924	1.30 (1.14)	0.253
Skull HU per 1 SD decrease	4.58 (10.01)	0.648	31.59 (69.47)	0.649	−17.18 (13.93)	0.219	2.84 (53.36)	0.957
Time, y	128.08 (3.41)	<0.001	89.28 (30.42)	0.003	125.02 (4.66)	<0.001	105.70 (26.52)	<0.001
Skull HU per 1 SD decrease × Time	**14.66 (3.42)**	**<0.001**	79.60 (25.98)	0.002	**21.99 (4.41)**	**<0.001**	0.70 (5.96)	0.907
Age × Time			0.65 (0.45)	0.155	–	–	–	–
Age × Skull HU per 1 SD decrease	–	–	−0.29 (1.05)	0.781	–	–	–	–
Age × Skull HU per 1 SD decrease × Time	–	–	−**1.04 (0.39)**	**0.008**	–	–	–	–

Results of linear mixed models for levodopa-equivalent doses while controlling for disease duration, DAT availability in the posterior putamen, BMI, and WMHs.

BMI, body mass index; DAT, dopamine transporter; HU, Hounsfield units; WMHs, white matter hyperintensities. Bold indicates *p* < 0.05.

## Discussion

Baseline skull HU was used as a surrogate marker for osteoporosis in this study. Although T-score measured at the lumbar spine or proximal femur using dual-energy x-ray absorptiometry (DXA) is the gold standard for osteoporosis diagnosis, a previous study suggested that HU value measured in CT scans could be a reliable alternative method for bone mineral density and osteoporosis assessment (Pompe et al., 2016). This is especially true, in HU values obtained from the trabecular region of interest in the lumbar vertebrae, showing good reliability and sensitivity in osteoporosis detection, as defined based on the DXA T-score (Jang et al., 2019). Furthermore, several studies have shown that skull HU was significantly associated with T-score, allowing its usage as an opportunistic screening tool for osteoporosis (Na et al., 2018). Similarly, in the present study, we demonstrated that skull HU had a strong positive correlation with L1 HU, consistent with previous findings. Based on this, we investigated the associations of low skull bone density with nigrostriatal dopaminergic degeneration and the clinical course of drug-naïve female PD patients. The major findings were as follows. First, there was no association between skull HU and baseline DAT availability. Second, baseline skull HU was a significant predictor of the development of LID and wearing-off in relatively younger PD patients; however, this association was not observed in older PD patients. Lastly, baseline skull HU was closely associated with longitudinal LED in younger PD patients. Together, these findings suggest that baseline skull HU is closely associated with motor prognosis in drug naïve female PD patients in an age-dependent manner.

To the best of our knowledge, this is the first study to explore whether early-stage bone density was associated with baseline nigrostriatal dopaminergic neuronal density in female PD patients. Although the univariate linear regression analyses showed that lower skull HU values were associated with lower baseline DAT availability in the caudate, anterior putamen, and ventral striatum, these associations disappeared on multivariate linear regression analysis after adjusting for covariates. This was an unexpected result, considering the common pathomechanisms between osteoporotic bone density and dopaminergic neural loss. Previous studies have shown contradictory results regarding the association between osteoporosis and parkinsonian motor deficits that are closely coupled with dopaminergic neuronal density. Two studies showed the association between bone density and motor deficits in PD patients (Bezza et al., 2008; Kamanli et al., 2008), however, these studies had no adjustment for confounding factors. Moreover, Lorefält et al. (2007) failed to demonstrate significant relationship between bone mineral density and UPDRS-III score. Taken together, it appears that bone density is not a critical determinant of dopaminergic neural loss at the time of PD diagnosis. In postmenopausal osteoporotic patients, the detrimental effect of estrogen deficiency (Leranth et al., 2000) on nigrostriatal dopaminergic neurons could be counteracted with higher levels of osteocalcin (Kalaiselvi et al., 2013; Guo et al., 2018; Shan et al., 2019), wherein this offset effect may become gradually faint over time, since the deleterious effect of a diminished estrogen level exceeds the influence of osteocalcin (Kim et al., 2015; Jung et al., 2016). Given the complex relationship between estrogen, osteocalcin, bone mass, and nigrostriatal degeneration in PD, there is a need for its elucidation in future studies.

We found that baseline skull HU was not a significant predictor of the development of LID and wearing-off, but it was a significant predictor of longitudinal LED increase. Since skull HU was significantly correlated with age, and the relationship between aging and osteoporosis has been well established, we proceeded to perform an interaction analysis in the longitudinal model and found that there was a significant interaction effect between skull HU and age in the statistical model. Interestingly, subgroup analyses using the median split of age showed that only the patients with lower skull HU values had a greater risk of developing LID and wearing-off, and required more longitudinal LED over time in the relatively younger age group. Considering that the development of LID or wearing off is closely coupled with pre-and post-synaptic dopaminergic factors (Bezard et al., 2001; Cenci and Lundblad, 2006) and LED changes are indirectly associated with disease severity (Ayala et al., 2017), these findings provided indirect evidence of an age-dependent detrimental effect of osteoporosis on the disease progression of PD. Although the exact mechanism is uncertain, the common pathomechanisms underlying osteoporosis and PD may contribute to disease progression. Autophagy dysfunction, mitochondrial dysfunction, and inflammation play an important role in PD pathogenesis and have also been linked with bone health (Wang et al., 2016; Wang et al., 2020). In terms of age- dependent manner, we can find a clue in the distinct pathophysiology between postmenopausal osteoporosis and senile osteoporosis. Postmenopausal osteoporosis occurs between 50 and 65 years of age and is primarily due to estrogen deficiency, whereas senile osteoporosis develops after 70 years of age and is mainly due to the deficiency of mineral and vitamin and increased bone turnover (Hawkins et al., 2021). Considering that estrogen has been established as a protective hormone in PD (Ragonese et al., 2006; Lee et al., 2019), the low bone density in younger PD patients of this study may have been attributed to estrogen deficiency, which consecutively leads to unfavorable motor prognosis. Meanwhile, osteoporosis in the old age group may most likely be senile osteoporosis, which is not relevant to estrogen hormone and may have no effect on PD prognosis.

In this study, we enrolled a relatively large number of consecutive female patients with *de novo* PD, which may have minimized patient-sampling biases. A relatively long follow-up duration could provide more appropriate information about the long-term motor prognosis in PD patients. However, this study has some limitations. First, we investigated our hypothesis on the basis of a strong correlation between skull HU and L1 HU in healthy controls. Thus, further studies should investigate the association of osteoporosis with prognosis of PD using T-scores measured *via* DXA, which was previously established as the gold standard for osteoporosis diagnosis. Analysis on trajectory of bone density (either by DXA or CT-derived skull bone density) according to levodopa treatment can also be a future research topic of interest. Also, because bone densities in other body parts were not available in female PD patients in the present study, future studies are needed to show that the relationship between skull HU and L1 HU maintains in PD patients. Second, we assessed clinical progression based on the longitudinal changes in LED, which may have not accurately reflected the motor status of PD patients. Moreover, as this was a retrospective study, LID and wearing-off were determined based on medical records, which precluded definitive conclusions. Third, considering that menopause is one of the greatest risk factors for osteoporosis (Ji and Yu, 2015), the lack of information regarding menopause status could have acted as a confounding factor, although we attempted to minimize this effect by enrolling female PD patients with age ≥50, which is above the median age of natural menopause (Park et al., 2018). Fourth, this study did not consider physical activity, which is also an important preventive factor in both osteoporosis and motor prognosis in PD patients (Castrogiovanni et al., 2016; Schenkman et al., 2018). Finally, although the effect of drugs used for osteoporosis on PD patients remains unclear, information on osteoporosis treatment was unobtainable in this study, which may act as an unmeasured confounder. Nevertheless, our study at least provided a novel proof-of-concept that systemic bone loss measured using skull bone HU in this study may be associated with motor prognosis in female patients with PD, which need to be validated in larger prospective studies with consecutive DXA testing.

In conclusion, the present study demonstrated that baseline skull HU, which is easily obtainable in DAT scan, could be a predictor of longitudinal motor prognosis in young female PD patients. Further studies investigating whether treatment for osteoporosis can alter the clinical course of PD are warranted.

## Data availability statement

The de-identified data that support the findings of this study are available from the corresponding author upon request. The data are not publicly available due to privacy or ethical restriction.

## Ethics statement

The studies involving human participants were reviewed and approved by Yonsei University Severance Hospital Institutional Review Board (IRB No. 4-2014-0637). Written informed consent for participation was not required for this study in accordance with the national legislation and the institutional requirements.

## Author contributions

SHJ and NH: conceptualization, data curation, formal analysis, investigation, methodology, resources, visualization, and writing—original draft. HSL: formal analysis and methodology. SH, Y-gL, and YL: data curation and investigation. YR and YHS: investigation and writing—review and editing. PHL: conceptualization, investigation, methodology, resources, writing—original draft, and writing—review and editing. All authors contributed to the article and approved the submitted version.

## Footnotes

1
http://www.r-project.org/
